# In Vitro Culture, Genetic Uniformity Assessment, and Biochemical Traits of *Plectranthus amboinicus* (Lour.) Spreng

**DOI:** 10.3390/plants15132061

**Published:** 2026-07-02

**Authors:** Ana-Maria Radomir, Ramona Stan, Andreea Elena Manolescu, Doina Clapa, George Adrian Peticilă, Dorin Ioan Sumedrea

**Affiliations:** 1 National Research and Development Institute for Biotechnology in Horticulture Stefanesti-Arges, 37 Bucharest-Pitesti Road, 117715 Stefanesti, Romania; radomir.anamaria@yahoo.com; 2 Faculty of Horticulture and Business in Rural Development, University of Agricultural Sciences and Veterinary Medicine of Cluj-Napoca, 3-5 Manastur St., 400372 Cluj-Napoca, Romania; doina.clapa@usamvcluj.ro; 3 Faculty of Horticulture, University of Agronomic Sciences and Veterinary Medicine of Bucharest, 59 Marasti Blvd., District 1, 011464 Bucharest, Romania; apeticila@gmail.com; 4 The Academy of Agricultural and Forestry Sciences “Gheorghe Ionescu-Sisesti”, 61 Marasti Blvd., District 1, 011464 Bucharest, Romania; dsumedrea@yahoo.com

**Keywords:** Indian borage, micropropagation, plant growth regulators, genetic fidelity, RAPD markers, total phenolic content, total flavonoid content

## Abstract

This study aimed to develop an efficient micropropagation protocol for *Plectranthus amboinicus* (Lour.) Spreng, evaluate the genetic fidelity of in vitro-regenerated plants using RAPD molecular markers, and perform a biochemical assessment of micropropagated material compared with acclimatized plants. For the initiation of in vitro cultures, explants (uninodal fragments) were inoculated on Murashige and Skoog (MS) medium without plant growth regulators (PGRs). Various plant growth regulator treatments were tested to evaluate the in vitro regenerative potential of the explants. The highest multiplication rate (3.43 ± 0.21 shoots per explant) was obtained on MS basal medium supplemented with 3 mg/L 6-benzylaminopurine (BAP) and 3 mg/L gibberellic acid (GA_3_), while the greatest shoot length (3.22 ± 0.22 cm) was achieved on MS medium containing 1 mg/L BAP and 3 mg/L GA_3_. Indole-3-butyric acid (IBA) (0.5 mg/L), applied alone or in combination with GA_3_ (3 mg/L), was the most effective treatment for inducing a well-developed root system, facilitating successful acclimatization of regenerated plants (100% survival rate). In vivo rooting of shoots simultaneously with acclimatization was also tested. The best results (100% rooting rate) were obtained using perlite or Jiffy peat pellets as the rooting substrate, and the shoots were treated with Radi Stim. Molecular analyses confirmed the genetic fidelity of the micropropagated plants. Biochemical analyses revealed higher levels of phenolic compounds and flavonoids in in vitro-cultured plants than in acclimatized plants. These results validate the micropropagation protocol morphologically, genetically, and biochemically, and highlight in vitro culture as an efficient approach for producing high-quality *P. amboinicus* plant material and for the sustainable production of valuable bioactive compounds.

## 1. Introduction

*Plectranthus amboinicus* (Lour.) Spreng is a perennial plant species in the *Lamiaceae* family, commonly known as Indian borage, native to eastern and southern Africa and naturally widespread in tropical and warm regions worldwide [1]. This plant is widely used in folk medicine and for culinary purposes due to its medicinal properties and an oregano-like aroma [2].

*P. amboinicus* contains large amounts of bioactive compounds, including phenols, flavonoids, tannins, alkaloids, glycosides, amino acids, terpenoids, and sesquiterpenoids [3,4,5]. The main constituents of the essential oil in its leaves are: carvacrol, thymol, β-caryophyllene, α-humulene, γ-terpinene, p-cymene, α-terpineol, and β-selinene [6,7,8,9]. Due to the biological activities exhibited by these biochemical constituents (antioxidant, antimicrobial, anti-inflammatory, analgesic, antiepileptic, and antitumor activities), this plant is used to treat conditions such as asthma, cold, cough, fever, headache, constipation, and skin diseases [1,10,11].

*P. amboinicus* rarely flowers and produces seeds; therefore, vegetative propagation by stem cuttings is the most commonly used propagation method [1]. However, this approach provides a relatively low multiplication rate and may facilitate the transmission of systemic pathogens from the mother plant. Furthermore, when propagated plants are used for metabolite extraction, various factors can influence and limit the yield. These factors include tissue maturity, geographical location of the growing region, environmental conditions, water availability, and disease impact [12,13].

To overcome these limitations and to enable rapid large-scale propagation, tissue culture techniques have been widely employed as an alternative method. In vitro culture allows year-round plant production under controlled environmental conditions and facilitates the rapid generation of large numbers of uniform plantlets from limited starting material. In addition, the use of aseptic culture conditions contributes to the production of pathogen-free planting material and supports the conservation and sustainable utilization of valuable medicinal plant resources [14,15,16]. The success of micropropagation largely depends on the type and concentration of plant growth regulators (PGRs) used in the culture medium. Cytokinins are widely used to stimulate axillary bud development and shoot proliferation, while auxins contribute to root development [17,18,19,20]. Several studies have reported that the combination of gibberellic acid (GA_3_) with cytokinins promotes shoot elongation, improves internode development, and facilitates the production of morphologically normal shoots suitable for subsequent rooting and acclimatization. GA_3_ has also been frequently used in combination with auxins to promote root cell elongation and the development of a vigorous root system [21,22,23,24]. Therefore, evaluating different combinations of cytokinins or auxins with GA_3_ is important for establishing an efficient regeneration protocol for *P. amboinicus*.

Although direct organogenesis is generally associated with a lower frequency of somaclonal variation than indirect regeneration via callus, genetic changes can still occur during in vitro culture. Therefore, early assessment of genetic uniformity is recommended to confirm clonal fidelity and to validate the suitability of the propagation protocol for the production of true-to-type plants [25,26]. Currently, molecular markers are valuable biological tools for analyzing the genetic fidelity of in vitro regenerated plants [27]. Among DNA molecular markers, RAPD, ISSR, and SSR are effective in detecting genetic variation and validating clonal fidelity [26,28]. RAPD markers were selected in the present study because they are technically simple, cost-effective, do not require prior sequence information, and have been successfully applied to assess clonal fidelity in numerous plant species regenerated by tissue culture. Although ISSR and SSR markers can provide higher reproducibility and resolution, RAPD analysis remains a widely accepted and efficient approach for preliminary evaluation of genetic uniformity, especially in species with limited genomic resources [26,28,29].

In addition to the shoot regeneration process and biomass production, numerous elements under in vitro conditions, including light, elicitors, and PGRs, can influence the synthesis of secondary metabolites [30,31,32]. Phenolic compounds have emerged as potential targets for in vitro culture, which not only creates optimal circumstances for their production but also encourages increased accumulation and excretion of secondary metabolites by biosynthetic cells or tissues [33]. Phenolic compounds and flavonoids are important for plant defense and stress adaptation, and are often used as indicators of biochemical stability in medicinal plants [34]. The evaluation of the total content of phenolic compounds and flavonoids, both in in vitro grown plants and in acclimatized plants, provides information about the impact of micropropagation on phytochemical characteristics and biomass quality [35,36,37].

Although previous studies have reported micropropagation protocols for *P. amboinicus*, information integrating propagation efficiency, genetic fidelity, and biochemical characteristics of in vitro-derived biomass remains limited. Therefore, the present study combined morphogenetic, molecular, and biochemical approaches to provide a comprehensive evaluation of micropropagated *P. amboinicus* plants.

This study aimed to develop an efficient micropropagation protocol for *P. amboinicus*, evaluate the clonal fidelity of in vitro regenerated shoots using RAPD molecular markers, and perform a preliminary assessment of total phenolic and flavonoid contents in in vitro-cultured and acclimatized plants to evaluate the potential of in vitro-derived biomass as a source of bioactive compounds.

## 2. Results and Discussion

### 2.1. In Vitro Propagation

#### 2.1.1. In Vitro Culture Initiation Stage

The observations made four weeks after the initiation of cultures revealed that the inclusion of the *P. amboinicus* species in the in vitro culture system does not pose any particular problems; the use of CaCl_2_O_2_ (6%, 8%, and 10%) for the sterilization of explants proved effective. The percentage of initiation, the percentage of necrotic explants, and the contamination rates varied depending on the concentration of the CaCl_2_O_2_ solution used for disinfection, but there were no significant differences between the experimental variants.

**Figure 1:**
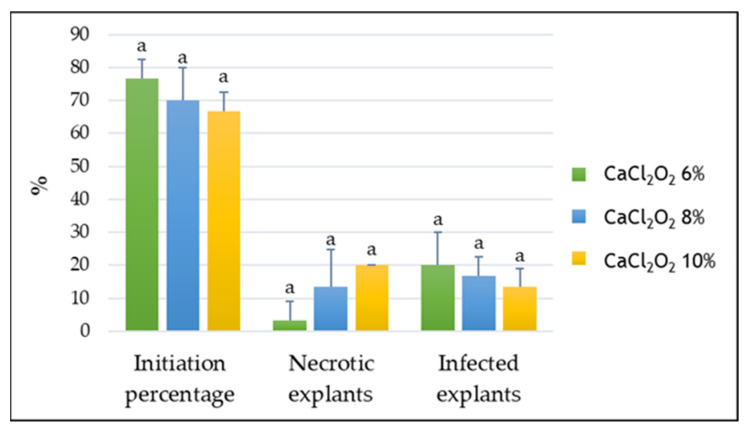
Influence of sterilization method on the in vitro regenerative potential of *Plectranthus amboinicus* (Lour.) Spreng explants at the culture initiation stage. Values shown are means ± SD. The same lowercase letter indicates no significant differences between treatments for each parameter evaluated, according to the Tukey HSD test (*p* < 0.05).

The initiation percentage ranged from 66.67% to 76.67% for the tested CaCl_2_O_2_ concentrations, while contamination ranged between 13.33% and 20%, and necrosis ranged between 3.33% and 20%. Treatment with 6% CaCl_2_O_2_, which provided the most favorable results, was associated with an initiation percentage of 76.67%, a contamination rate of 20%, and a necrosis rate of 3.33% (Figure 1).

Previous studies have shown that optimal sterilization conditions are species-dependent, since increasing disinfectant concentrations may enhance disinfection efficiency while adversely affecting explant viability through phytotoxic effects [38,39,40]. Arumugam et al. [21] reported that gentle brushing of explants to remove particles retained by trichomes, followed by treatment with 70% (*v*/*v*) ethanol for 10 s and 40% (*v*/*v*) Clorox^®^ for 15–20 min, resulted in a 71.11% survival rate of *P. amboinicus* explants. In *P. edulis*, excessive concentrations of bleach have been associated with tissue damage [41].

Overall, the CaCl_2_O_2_ concentrations tested in the present study produced initiation, contamination, and necrosis values within relatively narrow ranges. Since successful in vitro establishment requires both viable and contamination-free explants, sterilization protocols should be optimized according to the specific requirements of the species and culture conditions. The present results contribute to defining suitable sterilization conditions for *P. amboinicus* and provide a basis for further refinement of the propagation protocol.

Regenerated plantlets at the in vitro culture initiation stage were subsequently used to test the morphogenetic response of *P. amboinicus* explants on Murashige and Skoog (MS) basal medium [42] with different hormonal formulas.

#### 2.1.2. In Vitro Multiplication Stage

The results obtained at this stage of in vitro culture showed that the type, concentration, and combination of PGRs significantly influenced both the number of shoots per explant and shoot length in *P. amboinicus*, confirming the strong dependence of morphogenetic responses on exogenous hormonal balance.

**Figure 2:**
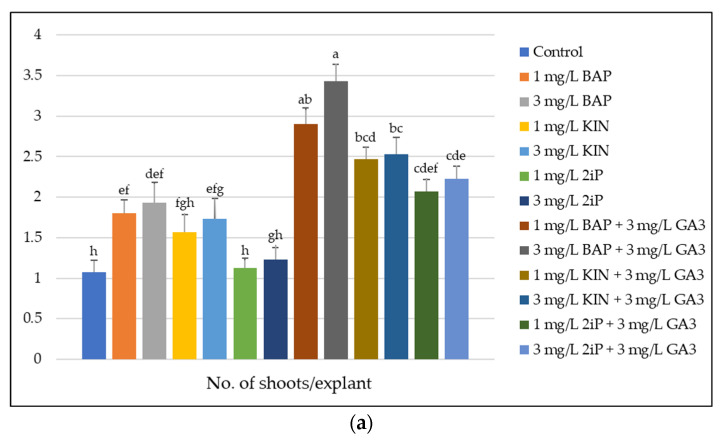

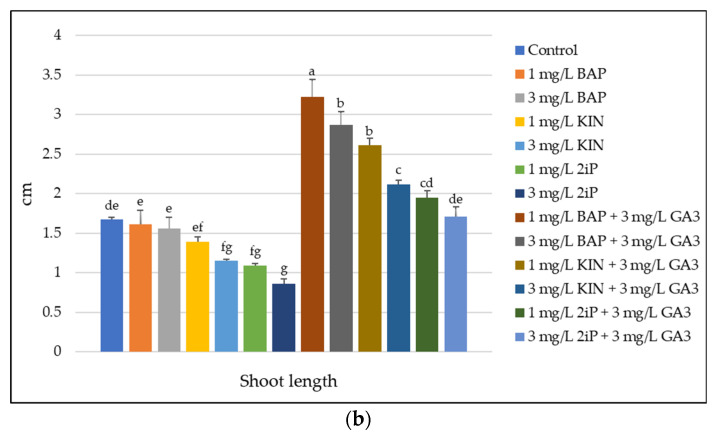
Influence of the type, concentration, and combination of PGRs on the number of shoots/explant (**a**) and on the shoot length (**b**) in the species *Plectranthus amboinicus* (Lour.) Spreng. Values shown are means ± SD. Different lowercase letters indicate significant differences between treatments, according to the Tukey HSD test (*p* < 0.05).

In the absence of PGRs (V0), explants produced only 1.07 ± 0.15 shoots/explant and a shoot length of 1.67 ± 0.03 cm, indicating a limited endogenous capacity for shoot proliferation and elongation (Figure 2a,b). This highlights the need for exogenous cytokinins for efficient shoot induction in *P. amboinicus* [38].

Among the cytokinins applied alone, BAP (6-benzylaminopurine) was the most effective for shoot induction, at a concentration of 1 mg/L (V1), resulting in 1.80 ± 0.17 shoots/explant, and at 3 mg/L (V2), resulting in 1.93 ± 0.25 shoots/explant (Figure 2a). These results are consistent with previous reports in which BAP exhibited strong organogenic responses in *P. amboinicus* and other *Plectranthus* species. Arumugam et al. [21] and Faisal and Alatar [43] reported that in *P. amboinicus*, BAP at a concentration of 0.5 mg/L and 1.13 mg/L (5 μM), respectively, was effective in shoot regeneration. Similar results were obtained in *P. edulis* by Kebede and Abera [41].

The addition of 3 mg/L GA_3_ improved both proliferation and shoot elongation in all cytokinin treatments, indicating a strong synergistic interaction between gibberellins and cytokinins.

The highest number of shoots (3.43 ± 0.21 shoots/explant) was recorded in variant V8 (3 mg/L BAP + 3 mg/L GA_3_), significantly higher than in all other treatments. In variant V7 (1 mg/L BAP + 3 mg/L GA_3_), a large number of shoots (2.90 ± 0.20 shoots/explant) was also produced (Figure 2a). These results demonstrate the benefit of combining cytokinin with gibberellin for maximizing shoot proliferation.

Interestingly, although variant V8 (3 mg/L BAP + 3 mg/L GA_3_) produced the highest number of shoots (3.43 ± 0.21 shoots/explant), the greatest shoot length was observed in variant V7 (1 mg/L BAP + 3 mg/L GA_3_) (3.22 ± 0.22 cm). In variant V8 (3 mg/L BAP + 3 mg/L GA_3_), shoot length was slightly lower (2.87 ± 0.17 cm), suggesting that a higher concentration of BAP associated with GA_3_ favors proliferation at the expense of elongation (Figure 2a,b). This result indicates that the optimal hormonal balance for maximizing shoot number may differ from that promoting shoot elongation. In this context, shoot length can be considered an indicator of vigor and potential performance in subsequent stages (e.g., rooting and acclimatization), but it does not fully define overall shoot quality, which also depends on other morphological and physiological traits. Similar trends have been reported in other micropropagation studies, where higher cytokinin concentrations increased multiplication rates while limiting shoot elongation [23,38].

A positive effect of combining cytokinins with GA_3_ on regenerative processes was also reported by Arumugam et al. [21]. In the study conducted by Sahoo et al. [24], the highest average shoot length (3.1 cm) was obtained on medium containing 3 mg/L BAP and 1 mg/L GA_3_, and the percentage of shoot regeneration was maximum (88%) on MS medium supplemented with 1 mg/L BAP, 5 mg/L NAA (α-naphthaleneacetic acid), and 1 mg/L GA_3_. In contrast to our results, there are studies in which the combination of cytokinin with auxin led to the best results in terms of shoot multiplication in *P. amboinicus*. Research by Faisal and Alatar [43] demonstrated that MS medium supplemented with 5 μM BAP and 2.5 μM NAA was found to be the most effective for shoot regeneration and multiplication. Fonseka and Katepearachchi [44] reported the highest shoot proliferation rate (75%) and the highest number of shoots per explant (7.8) on medium supplemented with 2 mg/L BAP and 0.1 mg/L NAA. The efficiency of combining cytokinin with auxin for shoot multiplication and elongation has also been reported in other studies conducted on this plant species [21,45].

In conclusion, for large-scale propagation of *P. amboinicus*, 3 mg/L BAP + 3 mg/L GA_3_ (V8) represents the optimal treatment due to the highest shoot multiplication rate. However, for obtaining elongated shoots suitable for rooting and acclimatization, 1 mg/L BAP + 3 mg/L GA_3_ (V7) provides a better balance between proliferation and shoot quality.

Overall, the results confirm that fine-tuning cytokinin type and concentration, together with strategic gibberellin supplementation, is essential for optimizing micropropagation protocols in *P. amboinicus*, in agreement with previous studies on this genus.

**Figure 3:**
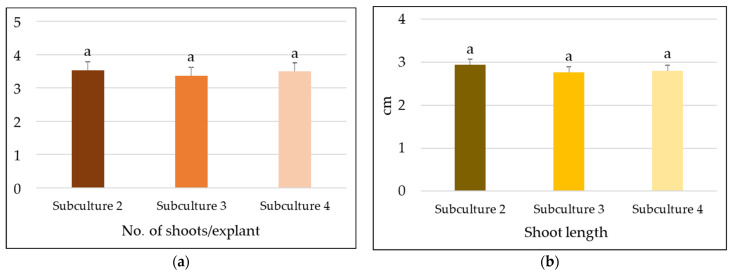
Influence of the number of subcultures on the number of shoots/explant (**a**) and on shoot length (**b**) in *Plectranthus amboinicus* (Lour.) Spreng cultured on MS culture medium supplemented with 40 g/L glucose, 32 mg/L NaFeEDTA, 7 g/L agar, 3 mg/L BAP, and 3 mg/L GA_3_. Values shown are means ± SD. The same lowercase letter indicates no significant differences between subcultures, according to the Tukey HSD test (*p* < 0.05).

In the present study, the in vitro regenerative potential of the studied species was also evaluated in three successive subcultures. The results obtained showed that the in vitro regenerative potential of *P. amboinicus* remained approximately at the same level throughout the subcultures performed. The number of subcultures did not have a negative effect on the two parameters evaluated. There were no statistically significant differences between the three subcultures in terms of the number of shoots/explant (Figure 3a) and shoot length (Figure 3b).

From a qualitative point of view, the biological material regenerated in vitro presented a normal morphology, without aspects of vitrification, necrosis, or callus differentiation.

#### 2.1.3. In Vitro Rooting Stage

In the present study, on many of the multiplication culture media used, some of the regenerated shoots also formed roots, resulting in a reduction in the time and expense required for in vitro rooting of shoots. We mention that, in the context of our study, the occurrence of spontaneous rooting did not negatively affect the efficiency of shoot multiplication, the quality or uniformity of regenerated plantlets. The spontaneous development of the root system on the multiplication medium may be related to the endogenous amount of auxin, as well as other intrinsic rooting factors. It has been estimated that spontaneous rooting of shoots can save costs of approximately 35 to 75% of the total budget required for in vitro regeneration [46].

The shoots that did not form roots on the propagation medium were transferred to the rooting medium.

The results obtained showed that the tested plant material presented a high natural capacity for root formation, the rooting rate on the control variant being 86.67%. A high rooting percentage (96.67%) on medium without PGRs was also obtained by Clapa et al. [47] on Driver and Kuniyuki Walnut (DKW) culture medium [48]. Arumugam et al. [21] also achieved better shoot rooting in vitro without the addition of PGRs in the culture medium.

**Figure 4:**
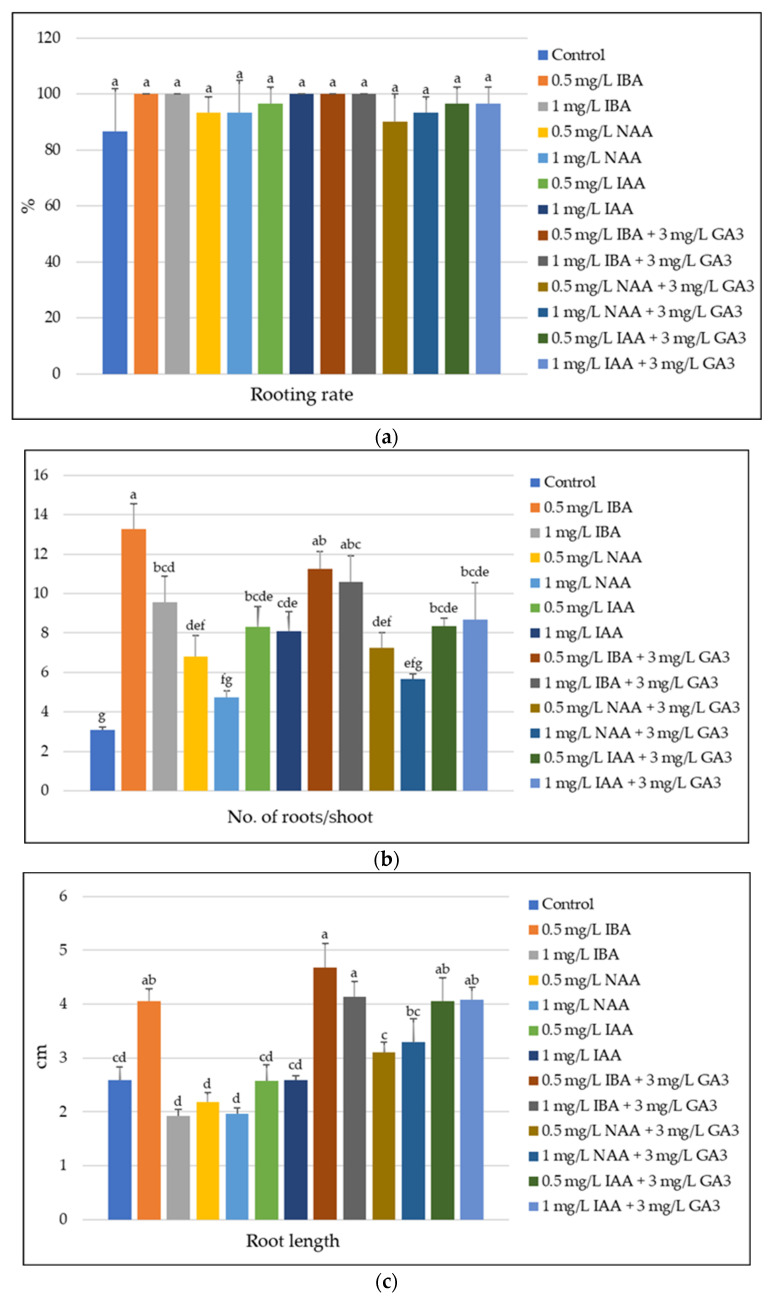
Influence of type, concentration, and combination of PGRs on in vitro rooting rate (**a**), number of roots/shoot (**b**), and root length (**c**) in *Plectranthus amboinicus* (Lour.) Spreng. Values shown are means ± SD. Different lowercase letters indicate significant differences between treatments, according to the Tukey HSD test (*p* < 0.05).

However, in the present study, although the Tukey HSD test (*p* < 0.05) indicated that phytohormone treatments did not significantly influence the rooting percentage, supplementing culture media with PGRs resulted in a rooting percentage of 100% in several variants (Figure 4a).

Among the auxins tested, IBA (indole-3-butyric acid) led to the best results, ensuring a maximum rooting percentage (100%) at both concentrations used (Figure 4a). These results are consistent with those reported in previous studies in *P. amboinicus*. Sahoo et al. [24] achieved 100% rooting on semisolid MS medium supplemented with 0.5 mg/L IBA. Similarly, Rahman et al. [45] reported that MS medium containing 1 mg/L IBA was the most effective treatment, also inducing 100% rooting. Faisal and Alatar [43] obtained a rooting rate of 93.7% on ½MS medium supplemented with 0.05 mg/L (0.25 μM) IBA, while Soliman [49] observed 75% rooting on hormone-free MS medium; however, supplementation with IBA increased rooting efficiency up to 100%.

The results obtained revealed a significant influence of the type and concentration of PGRs on the rhizogenesis process and in terms of the number and length of roots. On the control variant (V0), 3.10 ± 0.13 roots/shoot were formed, with an average length of 2.59 ± 0.25 cm, which confirms the lower rooting capacity in the absence of PGRs (Figure 4b,c). In contrast to our results, Fonseka and Katepearachchi [44] reported the longest roots in culture medium without PGRs.

Supplementation of culture media with auxins resulted in, in most cases, significant stimulation of root formation, compared to the control. Among the auxins tested, IBA proved to be the most effective, especially at the concentration of 0.5 mg/L (V1), at which the highest number of roots (13.28 ± 1.29) and an average length of 4.05 ± 0.24 cm were recorded (Figure 4b,c). These results are consistent with those reported by Hartmann et al. [50] and De Klerk et al. [51], who showed that IBA is one of the most effective auxins for inducing rhizogenesis due to its high stability. In contrast to our results, Fonseka and Katepearachchi [44] reported that the use of 0.5 mg/L IAA resulted in the highest number of roots/explant.

The combination of IBA with GA_3_ (V7 − 0.5 mg/L IBA + 3 mg/L GA_3_, V8 − 1 mg/L IBA + 3 mg/L GA_3_) led to the highest average lengths (4.68 ± 0.45 cm, respectively, 4.14 ± 0.28 cm) (Figure 4b,c). 

Statistical analysis of the differences between variants showed that IBA treatments, especially in combination with GA_3_, were significantly superior (*p* < 0.05) to the other treatments, both in terms of number and length of roots. Higher concentrations of auxins (1 mg/L) had an inhibitory effect, while GA_3_ showed a positive effect on root elongation, regardless of the auxin used.

It can be concluded that, in the case of the *P. amboinicus* species, IBA at a concentration of 0.5 mg/L, applied alone or in combination with GA_3_ (3 mg/L), represents the optimal treatment for the formation of a well-developed root system, an essential condition for the successful acclimatization of in vitro regenerated plants.

#### 2.1.4. Acclimatization Stage

- Acclimatization to Ex Vitro Conditions of In Vitro Rooted Shoots

The results of the assessment of the acclimatization capacity of *P. amboinicus* shoots rooted in vitro showed that they responded well to transfer to in vivo conditions. An acclimatization rate of 100% was recorded in both nutrient substrates used: Jiffy peat pellets and a mixture of peat, manure, and perlite (2:1:1). The high rate of ex vitro acclimatization of shoots confirms the efficiency of the micropropagation protocol developed for the production of healthy planting material.

Similar results, namely high acclimatization rates of *P. amboinicus* plantlets, have been reported by several authors: 75% [21], 90% [52], 94% [43], 95% [53], 98% [24,45], and 100% [44,47,49].

- In Vivo Rooting of Shoots Simultaneously with Acclimatization

**Figure 5:**
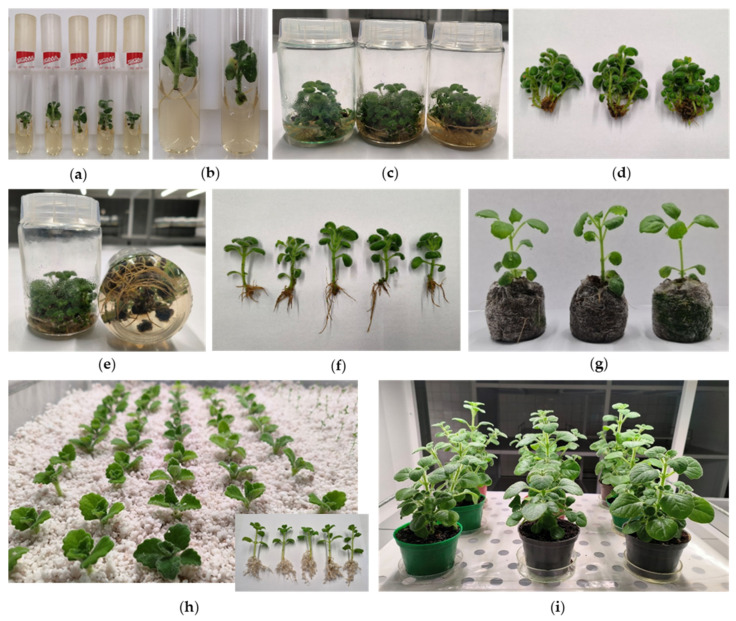
Aspects of in vitro culture of *Plectranthus amboinicus* (Lour.) Spreng: (**a**,**b**) explants in the initiation phase on MS medium without PGRs; (**c**,**d**) shoots multiplied on MS medium supplemented with 3 mg/L BAP and 3 mg/L GA_3_; (**e**,**f**) in vitro rooted shoots; (**g**) shoots rooted in vivo in Jiffy peat pellets; (**h**) shoots rooted in vivo in perlite; (**i**) acclimatized plants fortified in pots.

The results of the evaluation of the extent to which the composition of the rooting substrate and rooting stimulator influences the in vivo rooting of *P. amboinicus* shoots showed that this process occurred under optimal conditions. During this period, the shoots showed intense development of the root system, forming a significant number of roots (Figure 5g,h).

Statistical analysis of the data showed that the best results in terms of in vivo rooting rate (100%) were obtained, regardless of the substrate used, in the variants in which the shoots were treated with Radi Stim, but these were not statistically different from the results obtained in the other treatments (Table 1). Treating the base of the shoots with rooting stimulators favors cell division and the formation of adventitious roots, which allows shortening the rooting period and increasing the percentage of rooted shoots.

Simultaneously with the in vivo rooting of the shoots, their acclimatization to ex vitro conditions was also achieved, thus shortening the technological flow of obtaining planting material through micropropagation and considerably reducing the unit price of the plant.

The acclimatized plants were subsequently potted in a peat-based mixture for further growth and development. We note that the in vitro regenerated plants retained the morphological characteristics of the donor plants (Figure 5i).

Figure 5 shows aspects of the main stages of in vitro culture of *P. amboinicus*.

#### 2.1.5. Heatmap and Cluster Analysis

**Figure 6:**
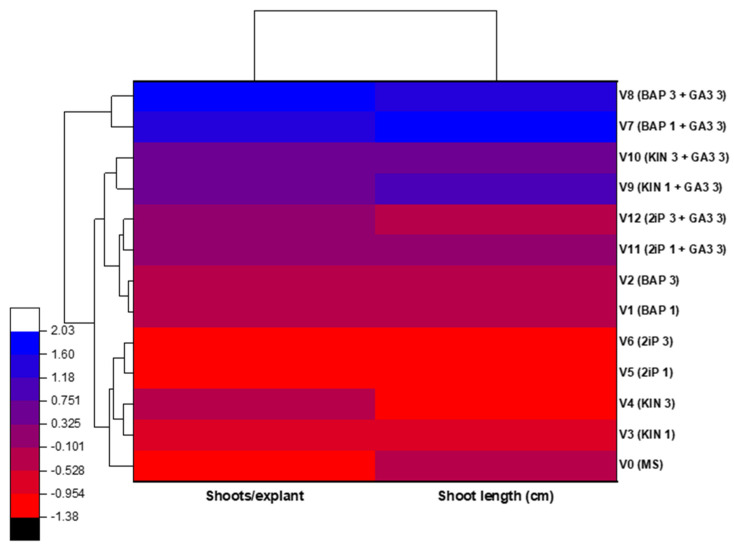
Heatmap of in vitro multiplication parameters in response to hormonal treatments in *Plectranthus amboinicus* (Lour.) Spreng. The heatmap provides an integrative visualization of the mean number of shoots per explant and shoot length (cm) obtained during the in vitro multiplication stage for the different hormonal treatments (V0–V12). Data were standardized by column using z-score normalization to allow comparison of parameters measured in different units. Similarity among treatments was assessed by hierarchical cluster analysis using Euclidean distance and the group average linkage method. Colors represent relative standardized values within each parameter, with blue indicating relatively higher values and red indicating relatively lower values. The associated dendrogram highlights the grouping of treatments based on their overall response patterns in shoot proliferation and elongation. Values represent the means of three biological replicates, each consisting of 10 explants (n = 30). Treatment codes (V0–V12) correspond to the hormonal variants detailed in Section 3.1.3.

The heatmap analysis provided an integrated overview of the response of *P. amboinicus* explants to the different hormonal treatments during the in vitro multiplication stage (Figure 6). Prior to heatmap construction, shoot number and shoot length were standardized using z-score normalization to enable comparison of variables measured in different units within a common analytical framework. Hierarchical clustering revealed distinct similarity patterns among the tested variants based on the combined response of shoot proliferation and elongation.

Treatments containing BAP, particularly in combination with GA_3_, formed a separate cluster characterized by a similar response profile across the evaluated multiplication parameters. Variants V7 and V8 formed a distinct cluster, reflecting a similar response profile across the evaluated multiplication parameters. In contrast, the control medium and treatments based on 2iP grouped together and exhibited comparatively lower multiplication performance.

Overall, the heatmap provides a complementary multivariate visualization of treatment similarities and response patterns by integrating multiple growth parameters into a single representation.

**Figure 7:**
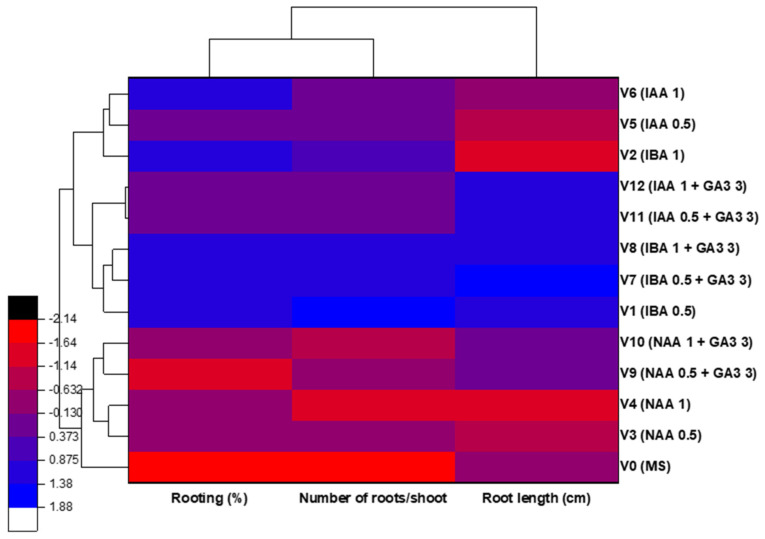
Heatmap of in vitro rooting parameters in response to hormonal treatments in *Plectranthus amboinicus* (Lour.) Spreng. The heatmap provides an integrated visualization of the in vitro rooting response of *P. amboinicus* explants to different hormonal treatments (V0–V12), based on the mean values of rooting percentage (%), number of roots per shoot, and root length (cm). Prior to heatmap construction, data were standardized by column using z-score normalization to allow comparison among parameters measured in different units. Similarity among treatments was assessed by hierarchical cluster analysis using Euclidean distance and the group average (UPGMA) linkage method. Colors represent relative standardized values within each parameter, with blue indicating relatively higher values and red indicating relatively lower values. The associated dendrogram highlights clustering of treatments according to overall rooting response patterns. Values represent the means of three biological replicates, each consisting of 10 explants (n = 30). Treatment codes (V0–V12) correspond to the auxin-based treatments described in Section 3.1.4.

The heatmap analysis also provided an integrated overview of the in vitro rooting response of *P. amboinicus* to the different hormonal treatments by simultaneously considering rooting percentage, number of roots per shoot, and root length (Figure 7). Prior to heatmap construction, these parameters were standardized using z-score normalization to allow comparison of variables measured in different units within a common analytical framework. Hierarchical clustering revealed distinct similarity patterns among the tested variants based on the combined rooting response.

Treatments containing IBA, particularly when combined with GA_3_, clustered together, indicating similar rooting response profiles across the evaluated parameters. In contrast, the control medium and treatments based on NAA or IAA generally grouped separately and exhibited comparatively lower rooting performance. Although the rooting percentage was high across most treatments, the heatmap clearly emphasized qualitative differences in root system development rather than simple root initiation.

Overall, the heatmap provides an integrated visualization of rooting response patterns among auxin-based treatments by simultaneously considering multiple morphological parameters.

### 2.2. Genetic Uniformity Assessment of In Vitro Regenerated Plants

RAPD analysis used to assess the genetic fidelity of in vitro propagated *P. amboinicus* plants generated clear and reproducible amplification profiles for all samples analyzed, including the mother plant, in vitro regenerated plants, and acclimatized plants (ex vitro). A total of 46 DNA fragments were amplified using the five selected RAPD primers (Table 2).

The size of amplified fragments ranged between 300 and 3000 bp, depending on the primer used. The number of bands produced by each primer varied between 8 and 10, with an average of 9.2 bands per primer.

The amplification profiles obtained with all primers showed monomorphic banding patterns across all analyzed samples. The banding patterns of the in vitro regenerated plants and acclimatized plants were identical to those of the mother plant, indicating the absence of detectable genetic variation during the micropropagation process.

**Figure 8:**
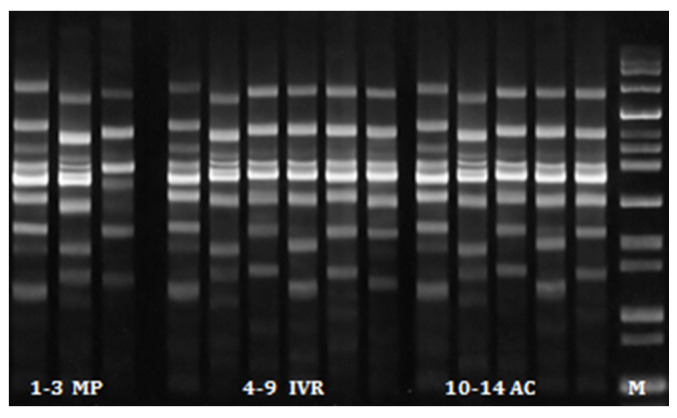
Representative RAPD electrophoretic profiles obtained with primer *OPA-1*. Lanes/bands: 1–3–mother plant (MP); 4–9–in vitro regenerated plants (IVR); 10–14–acclimatized plants (ex vitro) (AC); M–DNA ladder.

The monomorphic profiles observed in this study confirm the genetic uniformity of the regenerated plants of *P. amboinicus* after six in vitro subcultures, suggesting that the micropropagation and acclimatization procedures did not induce detectable genetic changes under the culture conditions employed (Figure 8).

Similar molecular approaches have been applied to other species of the genus *Plectranthus*. For example, RAPD markers were successfully used by Subositi et al. [29] to genetically characterize *P. scutellarioides*, demonstrating the usefulness of this technique for assessing genetic stability within plant populations.

The genetic stability of *P. amboinicus* plants grown in vitro was also confirmed by Faisal and Alatar [43] using SPAR markers, such as DAMD and ISSR, as well as flow cytometric analyses.

### 2.3. Biochemical Analysis

In the present study, total phenolic content (TPC) and total flavonoid content (TFC) of leaf extracts of in vitro plants were evaluated to assess the potential of in vitro-derived biomass as a source of bioactive compounds. Therefore, leaf extracts obtained from in vitro plants were compared with those from acclimatized ex vitro plants of *P. amboinicus*. As shown in Table 3, both total phenolic content (TPC) and total flavonoid content (TFC) were significantly higher in the biomass obtained in vitro than in acclimatized plants (Student’s t-test). Total phenolic content reached 55.091 µg GA g^−1^ fresh weight in potted plants and 76.364 µg GA g^−1^ fresh weight in in vitro-derived plants. Total flavonoid content showed values of 10.800 µg Q g^−1^ fresh weight in potted plants and 17.400 µg Q g^−1^ fresh weight in in vitro cultures (Table 3).

Similar results were obtained by Faisal et al. [52], who reported a higher total phenolic content in plants cultured in vitro compared to those grown ex vitro, while no significant difference was observed in terms of total flavonoid content between ex vitro and in vitro plants.

Taken together, the obtained results highlight not only the effectiveness of the proposed micropropagation protocol but also the quality of the regenerated plant material. Unlike previous reports focusing primarily on micropropagation efficiency, the present study combined morphogenetic evaluation, RAPD-based genetic fidelity assessment, and biochemical characterization of in vitro-derived biomass. This integrated approach provides additional information regarding both the quality of regenerated plants and their potential as a source of bioactive compounds.

## 3. Materials and Methods

### 3.1. In Vitro Propagation

#### 3.1.1. Culture Medium and Culture Conditions

In the experiments performed in this study, MS basal medium was used. In all in vitro culture stages, the culture medium was supplemented with 40 g/L glucose, 32 mg/L NaFeEDTA, and 7 g/L agar.

The culture media were sterilized by autoclaving at 120 °C for 20 min. Before autoclaving, the pH of the medium was adjusted to 5.6–5.8 with 1N KOH or 1N HCl.

The cultures were maintained in the growth chamber under controlled conditions of temperature (22–24 °C), photoperiod (16 h light/8 h dark), and light intensity (2500 lx-Philips Core Pro LED tube 1200 mm T8 15.5 W, 1800 lm Cool Daylight, Signify Romania S.R.L., Bucharest, Romania). 

#### 3.1.2. In Vitro Culture Initiation Stage

The explants used to initiate in vitro cultures consisted of uninodal fragments taken from shoots harvested from *P. amboinicus* mother plants grown in pots under greenhouse conditions. The mother plants used in the experiment were provided by the Plant Genetic Resources Bank Buzau, Romania. The shoots were defoliated, fragmented, and first washed with tap water, after which they were sterilized with CaCl_2_O_2_ in different concentrations: 6%, 8%, and 10% (*w*/*v*). The sterilization time was 10 min. Subsequently, three rinses with sterile distilled water were performed to remove traces of sterilizing agent. We mention that no surfactant was added during the surface sterilization procedure. After disinfection of the plant material, explants were sampled and inoculated under aseptic conditions on MS medium without PGRs. Cultures were grown in 36 mL culture tubes (2 cm diameter, 15 cm length) with transparent polypropylene lids, each containing 10 mL of medium (one explant per culture tube). They were maintained under the environmental conditions described above.

Four weeks after culture initiation, the following parameters were calculated: the percentage of infected explants (explants contaminated with fungal and/or bacterial infections), the percentage of necrotic explants (uncontaminated but non-viable explants, burned by the disinfectant agent), and the percentage of initiation (viable explants that have regenerated shoots).

#### 3.1.3. In Vitro Multiplication Stage

To induce shoot proliferation, the uninodal fragments resulting from the division of regenerated shoots during the in vitro culture initiation phase were inoculated on MS medium supplemented with different types, combinations, and concentrations of PGRs. MS medium without PGRs was used as a control (Table 4).

Cultures were grown in 200 mL jars (5 cm diameter, 9 cm height) with transparent polypropylene lids, each containing 30 mL of medium (five explants per jar). They were maintained under the environmental conditions described above.

Four weeks after subcultivation, the number of shoots per explant and shoot length were evaluated as growth parameters.

To evaluate the in vitro regenerative potential of the studied species in successive subcultures, the regenerated biological material was cultured on MS culture medium supplemented with 40 g/L glucose, 32 mg/L NaFeEDTA, 7 g/L agar, 3 mg/L BAP, and 3 mg/L GA_3_. Three successive subcultures were performed at approximately four-week intervals. The parameters evaluated after each of the three subcultures were the number of shoots/explant and shoot length.

#### 3.1.4. In Vitro Rooting Stage

The shoots of 3-4 nodes regenerated during the in vitro multiplication phase were individualized and cultivated on several variants of rooting medium in which the type of auxin (IBA-indole-3-butyric acid; NAA-α-naphthaleneacetic acid; IAA-indole-3-acetic acid) and its concentration (0.5 and 1 mg/L) were varied. The combination of auxin with gibberellic acid (GA_3_) (3 mg/L) was also tested. ½MS medium without PGRs was used as a control (Table 5).

Cultures were grown in 200 mL jars (5 cm diameter, 9 cm height) with transparent polypropylene lids, each containing 30 mL of medium (ten explants per jar). They were maintained under the environmental conditions described above.

Four weeks after the initiation of the experiment, the following parameters were calculated: rooting rate, number of roots/shoot, and root length.

#### 3.1.5. Acclimatization Stage

- Acclimatization to Ex Vitro Conditions of In Vitro Rooted Shoots

In vitro rooted shoots were carefully removed from the culture vessels, and their root system was rinsed thoroughly under running tap water to remove residual agar medium and reduce the risk of contamination with phytopathogenic microorganisms. Subsequently, they were transplanted into Jiffy peat pellets and pots with a substrate composed of peat, manure, and perlite (2:1:1) and maintained in high-humidity conditions by covering them with polyethylene film. During acclimatization, plantlets were regularly sprayed with water to prevent dehydration. Two to three weeks after transfer to ex vitro conditions, when the plants began to grow, the humidity was gradually reduced by increasing the daily aeration time by uncovering the plants, allowing them to adapt to natural environmental conditions. The appearance of the first ex vitro leaves signaled the end of the acclimatization phase.

Four weeks after the start of the experiment, the acclimatization percentage was assessed.

- In Vivo Rooting of Shoots Simultaneously with Acclimatization

To reduce the costs associated with in vitro culture, but also to reduce the production period of planting material through micropropagation, the possibility of in vivo rooting of *P. amboinicus* shoots simultaneously with acclimatization was also tested.

To initiate the experiment, shoots 3–4 cm long, regenerated during the in vitro multiplication phase, were used. The bases of the shoots were washed with water to remove remaining traces of agar, which may favor the occurrence of infections upon their transfer to in vivo conditions. The rooting substrate used was represented by perlite (placed in trays) and Jiffy peat tablets, and rooting stimulation was achieved by using the biopreparation for rooting herbaceous cuttings, Radi Stim No. 1, in powder form. We mention that Radi Stim No. 1 is a commercial rooting formula used according to the manufacturer’s recommendations, but detailed information about its auxin composition is not publicly available.

During the experiment, the shoots were provided with optimal rooting conditions, a temperature of 22–25 °C, and high atmospheric humidity (85–90%). Airing and spraying with water were carried out daily, and when the shoots formed 1–2 new leaves, the humidity was gradually reduced. Thus, the photosynthesis process intensified, so that along with rooting, the shoots also acclimatized.

Four weeks after the start of the experiment, the percentage of rooted shoots in vivo was calculated.

The acclimatized plants were then transplanted into pots in a peat-based mixture for further fortification and development.

### 3.2. Genetic Uniformity Assessment of In Vitro Regenerated Plants

#### 3.2.1. Plant Material

Leaf samples were collected from the mother plant, in vitro regenerated plants (after six in vitro subcultures), and acclimatized plants (ex vitro). Young and healthy leaves were selected for genomic DNA extraction to evaluate genetic variation among the analyzed plant materials.

#### 3.2.2. Genomic DNA Isolation

Total genomic DNA was isolated from approximately 0.1 g of fresh leaf tissue using the Qiagen DNeasy Plant Mini Kit (Qiagen, Hilden, Germany), following the manufacturer’s protocol. The quality and quantity of extracted DNA were determined using a UV–Vis spectrophotometer (Eppendorf, Hamburg, Germany) by measuring absorbance at 260 and 280 nm.

#### 3.2.3. RAPD-PCR Analysis

Random amplified polymorphic DNA (RAPD) analysis was performed to investigate genetic variation among the parent plant, in vitro regenerated plants, and acclimatized plants. Five RAPD primers were selected for their ability to produce clear and reproducible amplification patterns (Table 2).

The PCR reaction mixture (25 μL) consisted of genomic DNA template (25 ng μL^−1^)—2 μL, PCR master mix—12.6 μL, primer (20 μM)—0.8 μL, and nuclease-free water—9.6 μL.

PCR amplification was performed using a PCR 512 thermal cycler (Techne Ltd., Cambridge, UK) under the following conditions: initial denaturation at 95 °C for 3 min; 39 cycles of denaturation at 94 °C for 1 min; annealing at 36 °C for 60 s; extension at 72 °C for 2 min; final extension at 72 °C for 7 min. After amplification, 5 µL of each sample was loaded onto an agarose gel (2.0% agarose in TAE buffer) and stained with ethidium bromide at 50 V for 90 min. Migrated bands were visualized and photographed with the Gene Flash Syngene Bio Imaging system (Syngene, Cambridge, UK) under UV light. A Quick-Load Purple 100 bp DNA Ladder (New England Biolabs, Ipswich, MA, USA) was used to estimate the sizes of the amplified DNA bands.

### 3.3. Biochemical Analysis

Total phenolic content (TPC) and total flavonoid content (TFC) were determined spectrophotometrically in in vitro-cultured and acclimatized *P. amboinicus* plants. Fresh plant material (0.5 g) was extracted with acidified methanol (1% HCl), and the obtained extracts were used for biochemical analyses.

TPC was determined using the Folin–Ciocalteu method [54,55], with gallic acid as a standard, and the results were expressed in µg gallic acid equivalents per g fresh weight (µg GA/g FW). TFC was determined using the aluminum chloride colorimetric method, with quercetin as a standard, and the results were expressed in µg quercetin equivalents per g fresh weight (µg Q/g FW) [56,57].

The concentration of TPC and TFC was calculated using standard calibration equations derived from gallic acid and quercetin, respectively. For TPC, the calibration equation was y = 0.0011x + 0.1319, with R^2^ = 0.9981. For TFC, the calibration equation was y = 0.001x + 0.0313, with R^2^ = 0.9952, where y represents the absorbance and x represents the concentration of standard equivalents used for calibration.

Absorbance was measured using a UV–Vis spectrophotometer (PerkinElmer Inc., Shelton, CT, USA).

### 3.4. Statistical Analysis

The experiments were organized in 3 repetitions. For each experimental variant and each repetition, 10 explants were used for the in vitro phases (initiation, multiplication, and rooting) and for the in vivo rooting phase, and 30 explants for the acclimatization phase. Data were expressed as mean ± standard deviation (SD).

Statistical interpretation of data was performed using the SPSS 14.0 for Windows program. Differences between treatments were analyzed using one-way ANOVA with Tukey’s HSD (Honestly Significant Difference) post hoc test. Differences were considered statistically significant at *p* < 0.05.

For comparisons involving only two groups (in vitro and acclimatized plants), differences in total phenolic content (TPC) and total flavonoid content (TFC) were analyzed using Student’s *t*-test. Differences were considered statistically significant at *p* < 0.05.

### 3.5. Heatmap and Cluster Analysis

Heatmap analyses were performed using mean values of the evaluated parameters for the in vitro multiplication and rooting stages. Data were standardized by column (z-score), and hierarchical clustering was conducted using Euclidean distance and the group average method. Heatmaps were used as descriptive tools to visualize multivariate response patterns and similarities among treatments.

Heatmap and cluster analyses were performed using OriginPro 2021 (OriginLab Corporation, Northampton, MA, USA).

## 4. Conclusions

This study developed an efficient micropropagation protocol for *P. amboinicus*. The type, concentration, and combinations of PGRs significantly influenced the in vitro regenerative potential of explants. MS medium supplemented with BAP (1 or 3 mg/L) and GA_3_ (3 mg/L) was most effective for shoot proliferation, while IBA (0.5 mg/L), alone or combined with GA_3_ (3 mg/L), promoted optimal root development and successful acclimatization. In vivo rooting was also effective, allowing simultaneous rooting and acclimatization, which shortens the micropropagation process and reduces production costs. Molecular analyses confirmed the genetic fidelity of regenerated plants. Preliminary biochemical evaluation revealed higher total phenolic and flavonoid contents in in vitro-cultured plants compared to acclimatized plants, indicating enhanced secondary metabolism under in vitro conditions. These findings validate the developed micropropagation protocol as a reliable strategy for clonal propagation and germplasm conservation of *P. amboinicus*, with in vitro biomass serving as a potential source of phenolic compounds. Future studies should focus on identifying individual compounds using chromatographic techniques and applying more specific molecular markers, such as SSR and ISSR, for higher-resolution assessment of genetic fidelity.

## Figures and Tables

**Table 1:** Influence of culture substrate and rooting stimulator on the in vivo rooting capacity of *Plectranthus amboinicus* (Lour.) Spreng shoots. Values shown are means ± SD. The same lowercase letter indicates no significant differences between treatments, according to the Tukey HSD test (*p* < 0.05).

Rooting Substrate	Rooting Stimulator	In Vivo Rooting Rate (%)
perlite	-	96.67 ± 5.77 a
perlite	Radi Stim No. 1	100 ± 0.00 a
Jiffy peat pellets	-	93.33 ± 5.77 a
Jiffy peat pellets	Radi Stim No. 1	100 ± 0.00 a

**Table 2:** Primer sequences, number, and size range of monomorphic amplified RAPD bands detected in the analyzed samples.

Primer Name	Primer Sequence 5′-3′	Size Range of Bands (bp)	No. of Monomorphic Bands
*OPA-1*	CAGGCCCTTC	500–3000	9
*OPA-18*	AGGTGACCGT	400–3000	8
*OPB-1*	GTTTCGCTCC	300–1500	10
*OPC-2*	GTGAGGCGTC	400–1600	10
*OPD-2*	GGACCCAACC	300–1200	9
Total	-	-	46

**Table 3:** Total phenolic content (TPC) and total flavonoid content (TFC) in *Plectranthus amboinicus* (Lour.) Spreng samples obtained from in vitro cultures and potted plants. Values are presented as mean ± SD (n = 3). Different letters within the same row indicate significant differences according to Student’s *t*-test (*p* < 0.05).

Parameter	In Vitro Plants	Potted Plants
Total phenolic content (µg GA g^−1^ FW)	76.364 ± 0.167 a	55.091 ± 0.299 b
Total flavonoid content (µg Q g^−1^ FW)	17.400 ± 0.226 a	10.800 ± 1.942 b

**Table 4:** Experimental variants: in vitro multiplication of *Plectranthus amboinicus* (Lour.) Spreng shoots.

Variant	Basal Medium	PGRs (mg/L)			
		BAP	KIN	2iP	GA_3_
V0	MS	-	-	-	-
V1		1	-	-	-
V2		3	-	-	-
V3		-	1	-	-
V4		-	3	-	-
V5		-	-	1	-
V6		-	-	3	-
V7		1	-	-	3
V8		3	-	-	3
V9		-	1	-	3
V10		-	3	-	3
V11		-	-	1	3
V12		-	-	3	3

BAP-6-benzylaminopurine; KIN-kinetin; 2iP-2-isopentyl adenine; GA_3_-gibberellic acid; MS-Murashige and Skoog medium [42].

**Table 5:** Experimental variants: in vitro rooting of *Plectranthus amboinicus* (Lour.) Spreng shoots.

Variant	Basal Medium	PGRs (mg/L)			
		IBA	NAA	IAA	GA_3_
V0	½MS	-	-	-	-
V1		0.5	-	-	-
V2		1	-	-	-
V3		-	0.5	-	-
V4		-	1	-	-
V5		-	-	0.5	-
V6		-	-	1	-
V7		0.5	-	-	3
V8		1	-	-	3
V9		-	0.5	-	3
V10		-	1	-	3
V11		-	-	0.5	3
V12		-	-	1	3

IBA-indole-3-butyric acid; NAA-α-naphthaleneacetic acid; IAA-indole-3-acetic acid; GA_3_-gibberellic acid; MS-Murashige and Skoog medium [42].

## Data Availability

The original contributions presented in this study are included in the article. Further inquiries can be directed to the corresponding authors.
